# Pharmacokinetics and Interspecies Allometric Scaling of ST-246, an Oral Antiviral Therapeutic for Treatment of Orthopoxvirus Infection

**DOI:** 10.1371/journal.pone.0061514

**Published:** 2013-04-18

**Authors:** Adams Amantana, Yali Chen, Shanthakumar R. Tyavanagimatt, Kevin F. Jones, Robert Jordan, Jarasvech Chinsangaram, Tove′ C. Bolken, Janet M. Leeds, Dennis E. Hruby

**Affiliations:** 1 SIGA Technologies Inc., Corvallis, Oregon, United States of America; 2 Gilead Sciences Inc., Foster City, California, United States of America; Drexel University College of Medicine, United States of America

## Abstract

Plasma pharmacokinetics of ST-246, smallpox therapeutic, was evaluated in mice, rabbits, monkeys and dogs following repeat oral administrations by gavage. The dog showed the lowest T_max_ of 0.83 h and the monkey, the highest value of 3.25 h. A 2- to 4-fold greater dose-normalized C_max_ was observed for the dog compared to the other species. The mouse showed the highest dose-normalized AUC, which was 2-fold greater than that for the rabbit and monkey both of which by approximation, recorded the lowest value. The Cl/F increased across species from 0.05 L/h for mouse to 42.52 L/h for dog. The mouse showed the lowest V_D_/F of 0.41 L and the monkey, the highest V_D_/F of 392.95 L. The calculated extraction ratios were 0.104, 0.363, 0.231 and 0.591 for mouse, rabbit, monkey and dog, respectively. The dog showed the lowest terminal half-life of 3.10 h and the monkey, the highest value of 9.94 h. The simple allometric human V_D_/F and MLP-corrected Cl/F were 2311.51 L and 51.35 L/h, respectively, with calculated human extraction ratio of 0.153 and terminal half-life of 31.20 h. Overall, a species-specific difference was observed for Cl/F with this parameter increasing across species from mouse to dog. The human MLP-corrected Cl/F, terminal half-life, extraction ratios were in close proximity to the observed estimates. In addition, the first-in-humans (FIH) dose of 485 mg, determined from the MLP-corrected allometry Cl/F, was well within the dose range of 400 mg and 600 mg administered in healthy adult human volunteers.

## Background

Variola virus (VAR), the etiological agent for smallpox has been eliminated by the successful global vaccination campaign coordinated by the World Health Organization (WHO). However, recent concerns over the potential to weaponize this virus and the possibility of accidental introduction into the environment of this and other poxviruses such as the monkeypox virus (MPX) have spurred profound interest in the development of small-molecule therapeutics for the prevention and treatment of smallpox. Also, the absence of an active vaccination campaign, coupled with potential health risks associated with the existing vaccine regimen all render highly imperative, the development of a safe and effective antiviral therapeutic for use as a countermeasure against poxvirus infection.

**Figure 1:**
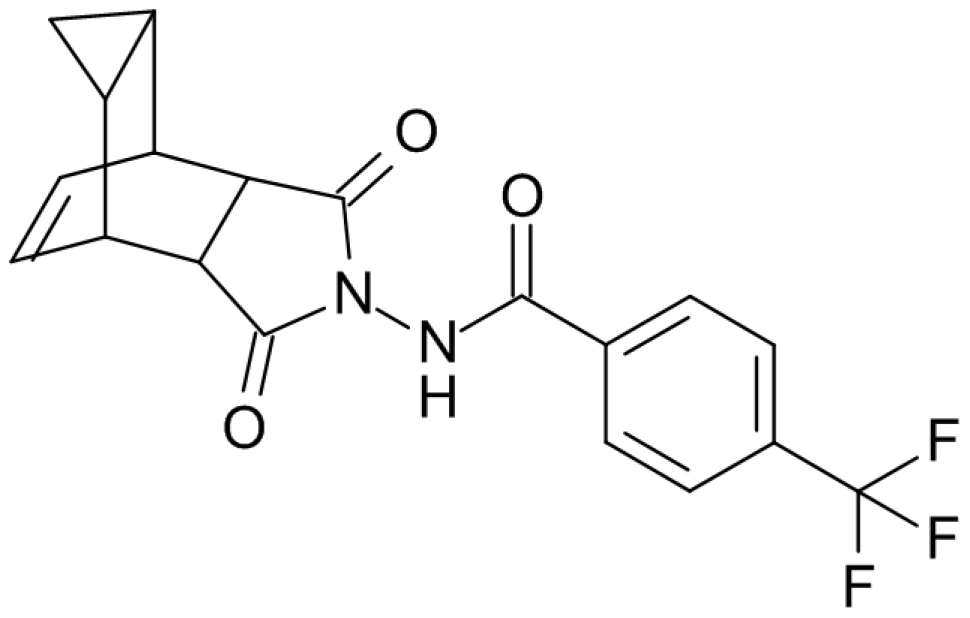
Molecular structure of ST-246.

ST-246 with molecular structure as shown in Figure 1, is an orally bioavailable molecule that is being developed by SIGA Technologies, for treatment of orthopoxvirus infections including smallpox. This compound has been shown to be extremely effective against a variety of orthopoxviruses, both *in vitro* and in multiple animal species [1]–[6].

Safety studies conducted in rodents, non-rodents and non-human primates at dose levels several fold greater than the efficacious dose indicate a high therapeutic index [6].

ST-246 acts by preventing the formation of extracellular virus particles and the molecular target is a highly conserved region of the F13L gene [7], [8].

This current study describes the oral plasma pharmacokinetics and utilizes traditional allometry to explore the interspecies relationship of the pharmacokinetics of ST-246 following oral single and repeat dose administrations. Preclinical data not only supports the assessment of drug safety, but is also useful in the determination of human pharmacokinetics for the selection of a dose for first-in-human trials. The ability to make such determinations is vital in optimizing the dose selection processes by minimizing time and cost in drug development [9], [10].

Mainly, two approaches, the physiologically-based model and allometric scaling are routinely utilized for interspecies pharmacokinetic scaling but with varying predictive success. Although the physiological model is mechanistic in nature and has been applied in the evaluation and extrapolation of drug pharmacokinetics [11]–[14], this approach is limited on the basis of cost, mathematical complexity, prolonged processing time and a high failure rate [15]. It is in view of these limitations that scaling by allometry, has gained broad application for interspecies pharmacokinetic extrapolation [16] and has been successfully applied to several small molecules. In this technique, preclinical pharmacokinetic parameters, plasma clearance (Cl/F) and apparent volume of distribution (V_D_/F) are scaled by the exponential function of body weight (BW) [17]–[19]. The predictive performance for this technique depends on factors such as data quality, number and type of species utilized in the extrapolation as well as the proper identification of the combination of physiological parameters and scaling factors that best relate to the disposition of a given molecule. Although, adequate extrapolations for Cl/F have been achieved based on the relation Y = AW^B^, this is not always the case as this model has the tendency to over-predict this parameter in some instances. In this regard, modified allometric scaling methods involving correction for animal brain weight [20] and maximum life span potential (MLP) [17], [21] are being utilized to improve prediction. The application of either one of these correction factors is determined by the “Rule of Exponents” (RoE) [20], [22]. The ROE states the following: A) if the exponent from the simple allometric equation is less than 0.50 or occurs between 0.55 and 0.70, then simple allometry is applied; B) if the simple allometry exponent is between 0.70 and 1.0, then the Cl/F X MLP correction approach is applied and C) if the simple allometry exponent is greater than 1.0, then the Cl/F X BrW correction approach is applied. MLP is calculated according to the following equation, MLP = 185.4 X (BrW) ^0.636^ X (BW)^−0.225^ [23].

Since oral administration is the preferred route for most drugs in clinical settings, the application of allometry for extrapolation to human pharmacokinetics will inevitably require the use of preclinical oral pharmacokinetics. Physiological factors, especially those associated with the gastrointestinal tract such as pH, blood flow rate and gastrointestinal transit time as well as the level of expression of enzyme systems involved in first-pass metabolism can determine the oral pharmacokinetic profiles for various molecules which in turn determine the outcome of interspecies scaling. Since these factors vary from species to species, preclinical species combination for scaling is crucial to the predictive success of this scaling technique.

In this study, we describe the oral plasma pharmacokinetics of ST-246 in animal species namely, BALB/c mice, New Zealand White Hra:(NZW)SPF albino rabbits, cynomolgus monkeys and beagle dogs as well as determine human oral plasma clearance (Cl/F), apparent volume of distribution (V_D_/F) and terminal half-life (t_1/2_) by interspecies allometric scaling.

## Materials and Experimental Procedures

### Materials

ST-246 (Tecovirimat: 4-trifluoromethyl-N-(3,3a,4,4a,5,5a,6,6aoctahydro-1,3-dioxo-4,6-ethenocycloprop[*f*]isoindol-2(1H)-yl)-benzamide) was synthesized by Pharmacore, High Point, North Carolina, USA and the identity confirmed by IR, MS, elemental analysis and NMR. Purity by HPLC was determined to be at least 95%. Other chemicals purchased from standard vendors include were of the highest purity possible.

### Animals for Pharmacokinetic Evaluation

Approximately six- to 10-week old BALB/c mice were received from Charles River Laboratories, Raleigh, North Carolina, USA and five- to six-months old New Zealand White Hra:(NZW)SPF albino rabbits were purchased from Covance Research Products, Inc., Kalamazoo, Michigan, USA.

Two- to three-year old cynomolgus monkeys were purchased from Covance Research Products, Alice, Texas, USA. Six- to eight-year beagle dogs were purchased from Marshall BioResources, U.S.A., Inc. North Rose, New York, USA.

All animals were housed in accordance with the National Institutes of Health Guide for the Care and Use of Laboratory Animals in individual cages placed in rooms fitted with unidirectional airflow systems, with temperature ranging from 64 to79°F, 61 to72°F, 64–84°F and 66–80°F for mice, rabbits, monkeys and dogs, respectively, and relative humidity ranging from 30 to 70% with a 12-hour light/dark cycle (6∶00 AM–6∶00 PM). Filtered tap water was available ad libitum via an automatic water system. The mice, rabbits, monkeys and dogs were fed a standard animal diet from PMI Nutritional International Inc., Saint Paul, Minnesota, USA, namely, Lab Diet® Certified Rodent Diet #5002, Lab Diet® Certified Rabbit Diet #5332 and Lab Diet® Certified Primate Diet #5048 and Lab Diet® Certified Canine #5007, respectively, and was available to the animals ad libitum. Food and water were withdrawn at periods during which dosing of the test article or blood draws for pharmacokinetic evaluation were conducted.

### Preclinical Pharmacokinetic Evaluation

The studies involving the mice, rabbits and monkeys were conducted at the MPI Research testing facility, Mattawan, MI, USA while that involving the dogs was conducted at Huntingdon Life Sciences, East Millstone, NJ, USA., in accordance with all Federal, State and Local guidelines for laboratory animal care and use. The use of these animals followed study protocols that were carefully reviewed and approved by the facility’s Institutional Animal Care and Use Committee (IACUC) as well as the IACUC for SIGA Technologies prior to the start of each study. In addition, all studies were conducted in compliance with the testing facility’s Animal Welfare Assurance (OLAW Assurance number A3181-01) filed with the National Institutes of Health. Veterinary care (vivarium rounds) by an American College of Laboratory Animal Medicine (ACLAM)-certified clinical veterinarian was available at all times throughout these studies at these facilities. In-life staff (technical operations and animal care) performed daily (AM and PM) husbandry procedures. Health monitoring, prophylactic and therapeutic treatments were conducted under the direction of a qualified veterinarian at these facilities. The veterinary staff monitored all animals and in the event where any of the animals exhibited any clinical signs, the appropriate treatment and supportive care were administered. Based on veterinary recommendation, animals experiencing severe or chronic pain/distress that could not be relieved were humanely euthanized without delay. Also, animals that made it to study termination were also humanely euthanized. The following methods of euthanasia that comply with the AVMA Guidelines on euthanasia were utilized. The mice were euthanized by anesthesia by carbon dioxide/oxygen inhalation followed by exsanguination. The rabbits were euthanasia by sodium pentobarbital administration followed by exsanguination. The monkeys were euthanized by ketamine sedation and by sodium pentobarbital administration followed by exsanguination. The dogs were euthanasia was by an intravenous overdose of sodium pentobarbital solution followed by exsanguination.

ST-246 was administered perorally by gavage as a suspension consisting of 1% hydroxypropyl methylcellulose (HPMC) in deionized water/0.5% Tween 80® for the plasma pharmacokinetic evaluation of this molecule. Verification of homogeneity stability and concentration of ST-246 the dose formulations was performed by HPLC with a UV system. Plasma concentration-time course data for non-compartmental pharmacokinetic evaluation in each of the species tested was generated based on the following study designs:

#### Pharmacokinetics in mice

Male and female BALB/c mice were given single daily administrations of ST-246 at dose of 2000 mg/kg, a dose volume of 10 mL/kg body weight for 28 consecutive days. Whole blood samples will be collected from the orbital sinus after carbon dioxide/oxygen inhalation at 1, 2, 3, 4, 6, 8, 10, 12 and 24 h following administration.

#### Pharmacokinetics in rabbits

In a seven-day repeat dose study, male and female New Zealand White Hra: (NZW) SPF albino rabbits received peroral single daily doses of ST-246 by gavage, at 100 mg/kg, at a dose volume of 10 mL/kg body weight. Plasma concentration of ST-246 was determined from whole blood samples collected via the ear vein at 0, 0.5, 1, 2, 3, 4, 5, 6, 8, 12 and 24 h post-dose administration.

#### Pharmacokinetics in monkeys

Male and female cynomolgus monkeys received ST-246 as single daily peroral administrations by gavage at100, at a dose volume of 10 mL/kg for 28 days. For the determination of plasma concentration of ST-246, whole blood samples were drawn via the femoral vein at 0, 1, 2, 3, 4, 5, 6, 8, 12 and 24 h following administration.

#### Pharmacokinetics in dogs

Male and female beagle dogs received ST-246 as single daily peroral administrations by gavage at dose of 30 mg/kg, at a dose volume of 10 mL/kg body weight for 7 consecutive days. Blood samples were drawn via the jugular vein at 0.5, 1, 2, 3, 4, 6, 8, 10, 12 and 24 h following administration.

Whole blood samples collected in all of these studies at the specified time points were immediately placed in tubes containing sodium heparin anticoagulant, stored on ice blocks until processed. The plasma generated from the blood samples were placed in clean tightly capped, pre-labeled plastic vials and frozen at approximately −70°C to await bioanalysis.

### Bioanalytical Reagents and Procedure

Quantitative analysis of the plasma samples at each time point was performed by liquid chromatography-tandem mass spectrometry (LC-MS/MS) (HPLC system: Agilent 1100 Series quaternary pump with on-line degasser, thermostatted well-plate autosampler and column compartment). Extraction of ST-246 from each sample was conducted by simple methanol (MeOH) protein precipitation in the presence of an isotopic internal standard for the preclinical samples. Briefly, 50 µL of uniformly mixed plasma was transferred into each well in a 96-well plate containing 5 µL working standard solution for calibration standards or working QC solution followed by the addition of a 150 µL of precipitation solution, MeOH containing the internal standard, ^13^C_4_-ST-246 (KXN-2327). This mixture was subsequently centrifuged at 4000 rpm for 5 minutes and a 100 µL aliquot was then added to the corresponding well in a fresh 96-well plate already containing 200 µL aliquot of the water/MeOH (50∶50 v/v) diluent. In the case of the clinical samples, solid phase extraction on an ISOLUTE®-96 Fixed Well Plate 200 mg SLE+ plate was performed. Briefly, 50.0 µL of each standard, sample, or blank was added to a v-bottom 96-well extraction plate. Then 50.0 µL of the working internal standard (WIS) was added to all wells except the double blanks. In place of WIS, 50.0 µL of Methanol/Water (50∶50 v/v) was placed in the double blanks wells. 100 µL of analytical grade water was then added to the appropriate wells of the 96-well plate. This was then thoroughly mixed and transferred to an ISOLUTE SLE®+ plate. Samples were then loaded onto the ISOLUTE SLE®+ plate with little or no vacuum and allowed to sit for approximately 5 minutes. The waste receptacle was replaced with the 96-well collection plate and 500 µL of methyl tertiary butyl ether (MTBE) added to all wells and eluted with minimal vacuum. An additional 500 µL of MTBE was added to all wells and eluted with minimal vacuum. The organic layer was evaporated under nitrogen at approximately 50°C. After the plate was dried, all samples were reconstituted with 500 µL of 0.05% NH_4_OH and 0.05% Acetic acid in MeOH/H2O (65∶35 v/v) and mixed. 50.0 µL of the reconstituted samples were subsequently diluted with 450 µL 0.05% NH_4_OH and 0.05% Acetic Acid in MeOH/H2O (65∶35 v/v) and thoroughly mixed.

Chromatographic separation was performed by means of a Phenomenex LUNA C18, 30×2 mm, 5 µm particle size column in 10 mM ammonium acetate (NH_4_AcO)/0.05% ammonium hydroxide (NH_4_OH) buffer in acetonitrile (ACN)/MeOH (50∶50 v/v) at a flow rate of 300 µL/min for the mobile phase. Analysis was conducted on Applied Biosystems/MDS SCIEX API 3000/4000 tandem liquid chromatography mass spectrometry (LC/MS/MS) using turbospray ionization in the negative ion mode. The mass spectrometer was tuned to the multiple reaction monitoring (MRM) scan mode to follow the m/z transitions, 375.1-282.9 and 340.9-248.9 for ST-246 and the internal standard, respectively. The linear calibration curve was calculated by the “least squares” method with a weighting factor of 1/X^2^ over the concentration range of 5.00–2000 ng/mL. The accuracy and precision of the bioanalytical method were within the acceptable criteria limits of ±15% RE of the nominal at all other concentrations and ±20% RE of the nominal for the LLOQ (5.00 ng/mL) [24].

### Plasma Concentration Data Processing

WinNonLin Professional Edition, Version 4.0 (Pharsight Corporation, Mountain View, CA, USA) was used to determine pharmacokinetic parameter estimates from the plasma ST-246 concentration data. The plasma exposure or area under the plasma concentration versus time curve (AUC) up to the time of the last quantifiable sample was calculated using the linear trapezoidal rule and was extrapolated to time infinity by the equation AUCt+(Clast/kel), where Clast is the concentration at the last quantifiable sampling time and kel is the elimination rate constant, determined by linear regression of the terminal log-linear phase of the concentration-time curve. Plasma clearance confounded by absolute oral bioavailability (Cl/F) was estimated based on the equation, Cl/F = Dose/AUC. The apparent volume of distribution confounded by absolute oral bioavailability (V_D_/F) was estimated as V_D_/F = Dose/(AUC×kel), where kel is the elimination rate constant. The terminal half-life (t_1/2_) was estimated by the relation t_1/2_ = In2/kel. The time (T_max_) to reach peak plasma concentration (C_max_) was determined directly from the observed plasma concentration versus time profiles. The calculated extraction ratio values were determined by the relation, Extraction ratio = Cl/F/Cardiac Output.

### Allometric Evaluation

Allometric scaling across species was performed to predict human plasma clearance (Cl/F) and apparent volume of distribution (V_D_/F). For our scaling process, simple allometry relating a given pharmacokinetic parameter to animal body weight (BW) was considered.

Each pharmacokinetic parameter, based on the simple allometric approach, was plotted against the body weight on a log-log scale based on the following power-based simple allometric equations for:

Simple Allometry
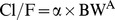


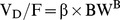



Corrected Allometry
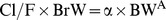


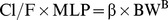



α&β = the allometric coefficients; A and B = Allometric Exponents.

BW = Body Weight; BrW = Brain Weight;

MLP = Maximum Life Span Potential.

Extrapolation to human pharmacokinetic parameters was based on a 70 kg body weight [17], [25]. In this evaluation, the Cl/F and V_D_/F were plotted against animal body weight on a log-log scale [16]. Linear regression analysis was then performed using GraphPad Prism 5.0, GraphPad Software, Inc. La Jolla, CA, USA, according to the logarithmic transformations of the simple allometric equations as shown below for:

Simple Allometry
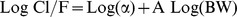


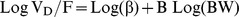



Corrected Allometry
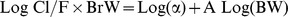


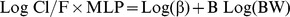



### First-in-Humans Dose Determination from Allometrically Scaled Human Clearance

A slight modification [26], of the pharmacologically guided approach, provided in the United State Food and Drug Administration (USDFA) draft guidance [27], was used to determine the first-in-humans (FIH) dose for ST-246 from the scaled human plasma clearance (Cl/F) value. In this approach [26], no safety factor was applied based on the assumption that the administered animal dose was significantly lower than a NOAEL, hence, the FIH dose expressed is as shown in the equation below:
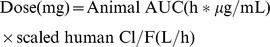
Where AUC is the lowest observed systemic exposure value among the species utilized in this study.

## Results

### Pharmacokinetics

**Figure 2:**
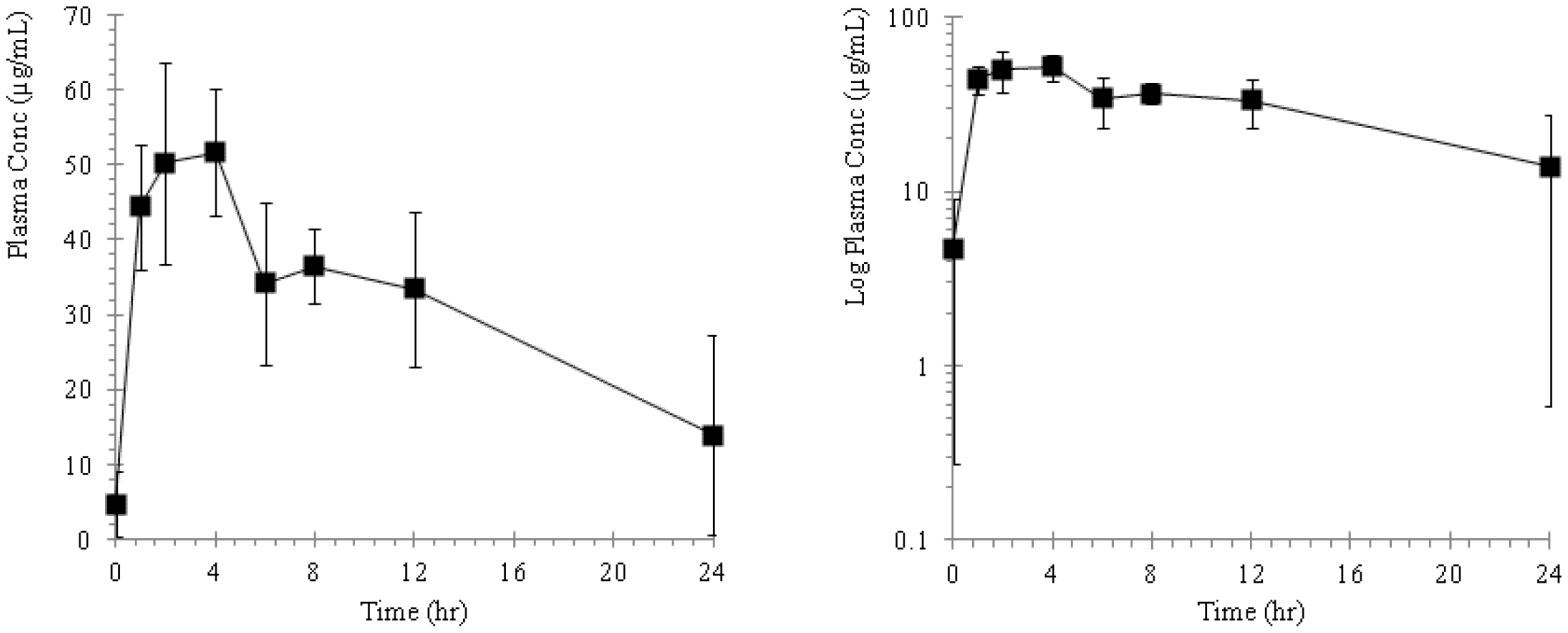
Plots of mean plasma concentration versus time profiles in BALB/c mice following repeat oral administrations of ST-246 by gavage at a dose of 2000 mg/kg for 28 consecutive days. The adjacent plot represents the corresponding semi-logarithmic plot.

**Figure 3:**
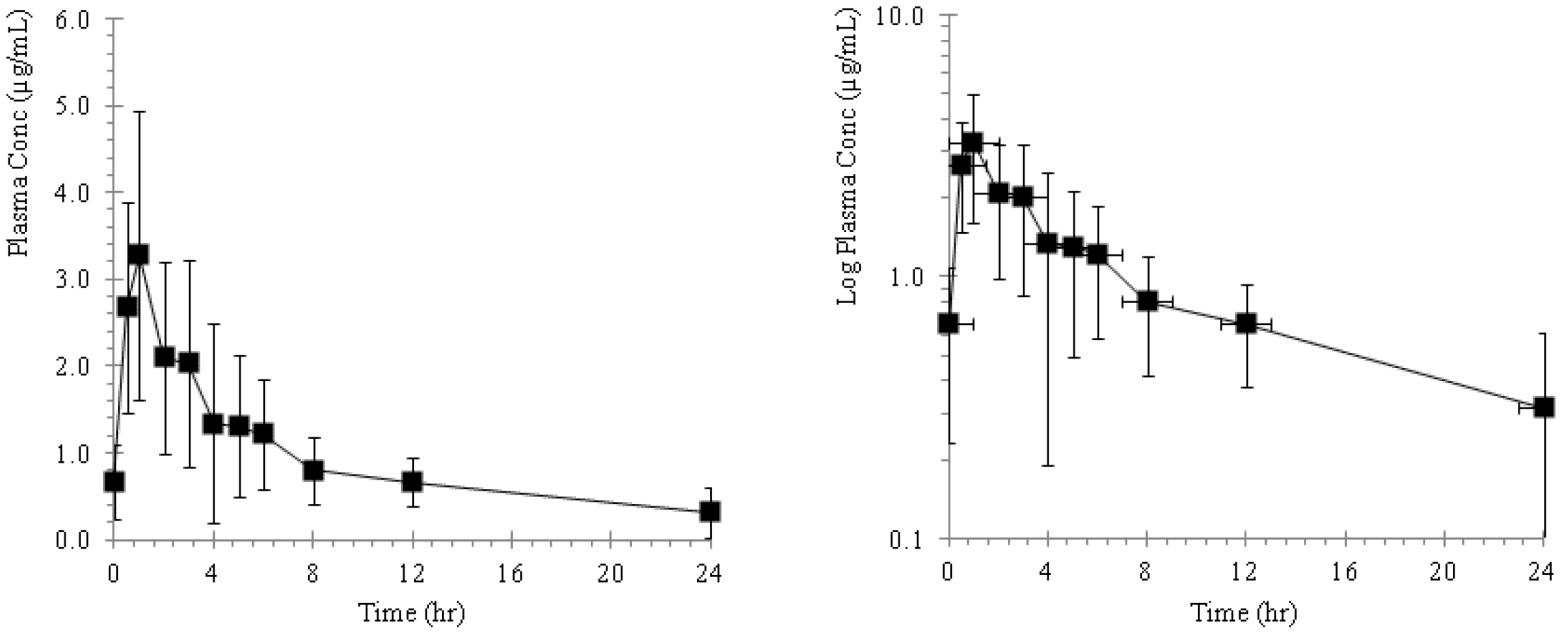
Plots of mean plasma concentration versus time profiles in New Zealand white Hra: (NZW) SPF albino rabbits following repeat oral administrations of ST-246 by gavage at a dose of 100 mg/kg seven consecutive days. The adjacent plot represents the corresponding semi-logarithmic plot.

**Figure 4:**
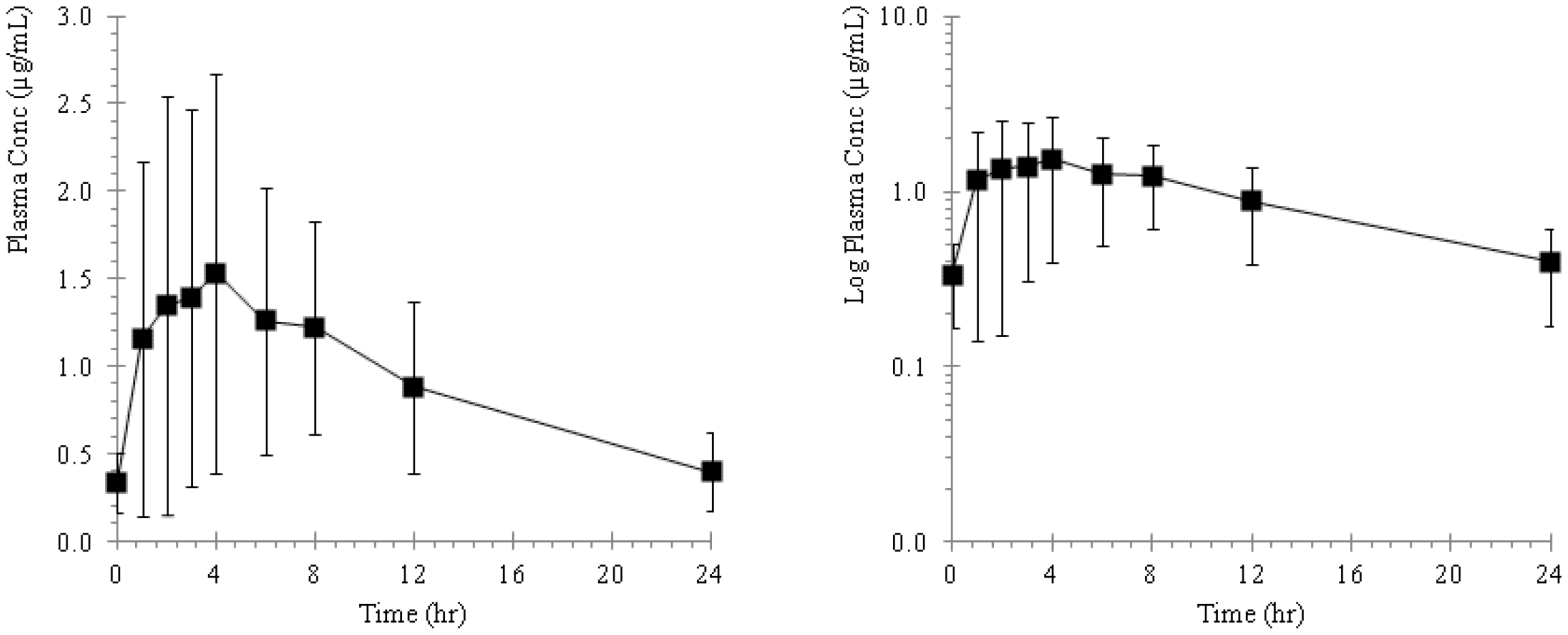
Plots of mean plasma concentration versus time profiles in cynomolgus monkeys following repeat oral administrations of ST-246 by gavage at a dose of 100 mg/kg for 28 consecutive days. The adjacent plot represents the corresponding semi-logarithmic plot.

**Figure 5:**
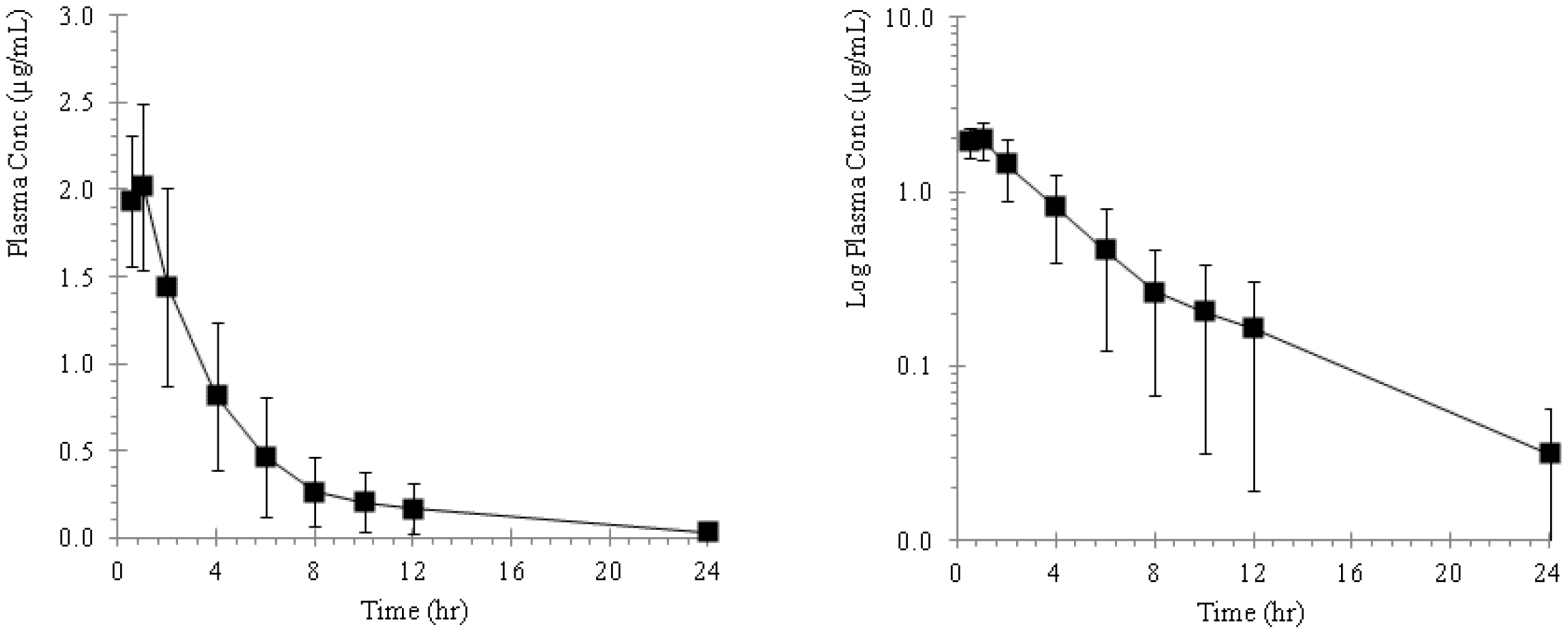
Plots of mean plasma concentration versus time profiles in beagle dogs, following repeat oral administrations of ST-246 by gavage at a dose of 30 mg/kg seven consecutive days. The adjacent plot represents the corresponding semi-logarithmic plot.

The plasma concentration of ST-246 in mice, rabbits, monkeys and dogs following repeat oral administration are presented as plasma concentration versus time profiles and illustrated in Figures 2, 3, 4, and 5 and the pharmacokinetic estimates (mean ±SD) are presented in Tables 1 and 2.

**Table 1:** Plasma pharmacokinetic estimates for BALB/c mice, New Zealand white Hra: (NZW) SPF albino rabbits, beagle dogs and cynomolgus monkeys following repeat oral dosing of ST-246 by gavage for 7 consecutive days in New Zealand white Hra: (NZW) SPF albino rabbits and beagle dogs and 28 consecutive days in BALB/c mice and cynomolgus monkeys.

Animal Species	Dose (mg/kg)	Cl/F (L/h)	V_D_/F (L)	t_1/2_ (h)	T_max_ (h)
Mouse	2000	0.05±0.01	0.41±0.17	5.81±0.86	3.00±1.41
Rabbit	100	11.54±3.42	129.02±90.35	7.27±3.23	1.50±1.41
Monkey	100	15.06±4.33	392.95±345.90	9.94±2.89	3.25±2.76
Dog	30	42.52±13.65	178.23±60.45	3.10±1.15	0.83±0.26

**Table 2:** Plasma pharmacokinetic estimates for BALB/c mice, New Zealand white Hra: (NZW) SPF albino rabbits, beagle dogs and cynomolgus monkeys following repeat oral dosing of ST-246 by gavage for 7 consecutive days in New Zealand white Hra: (NZW) SPF albino rabbits and beagle dogs and 28 consecutive days in BALB/c mice and cynomolgus monkeys.

Animal Species	Dose(mg/kg)	C_max_(μg/mL)	C_max_/Dose(μg/mL)/(mg/kg)	AUC(h*μg/mL)	AUC/Dose(h*μg/mL)/(mg/kg)	Extraction Ratio
Mouse	2000	53.03±8.11	0.027	858.60±241.89	0.429	0.104
Rabbit	100	2.91±1.66	0.029	20.07±8.82	0.201	0.363
Monkey	100	1.79±1.15	0.018	23.57±13.82	0.236	0.231
Dog	30	2.14±0.40	0.071	9.43±4.45	0.314	0.591

Extraction ratio = Cl/F/Cardiac Output.

As shown in Table 1, the lowest mean time to reach peak plasma concentration (T_max_) of 0.83 h was observed for the dog and the highest value of 3.25 h for the monkey. Also, a 2- to 4-fold greater dose-normalized peak plasma concentration (C_max_) was observed for the dog compared to the other species as listed in Table 2. The mouse showed the highest dose-normalized AUC, which was 2-fold greater than that for the rabbit and monkey both of which, by approximation showed the lowest dose-normalized AUC value, as shown in Table 2. The mean estimated plasma clearance (Cl/F) increased across species, from 0.05 L/h for mouse to 42.52 L/h for dog as displayed in Table 1. Also, as listed in Table 1, the dog showed the lowest mean terminal half-life of 3.10 h and the monkey, the highest value of 9.94 h. The lowest mean V_D_/F of 0.41 L was observed for the mouse and for the monkey, the highest estimate of 392.95 L as presented in Table 1. As listed in Table 2, the estimated extraction ratios for these animal species were 0.104, 0.363, 0.231 and 0.591 for mouse, rabbit, monkey and dog, respectively.

### Allometry

**Figure 6:**
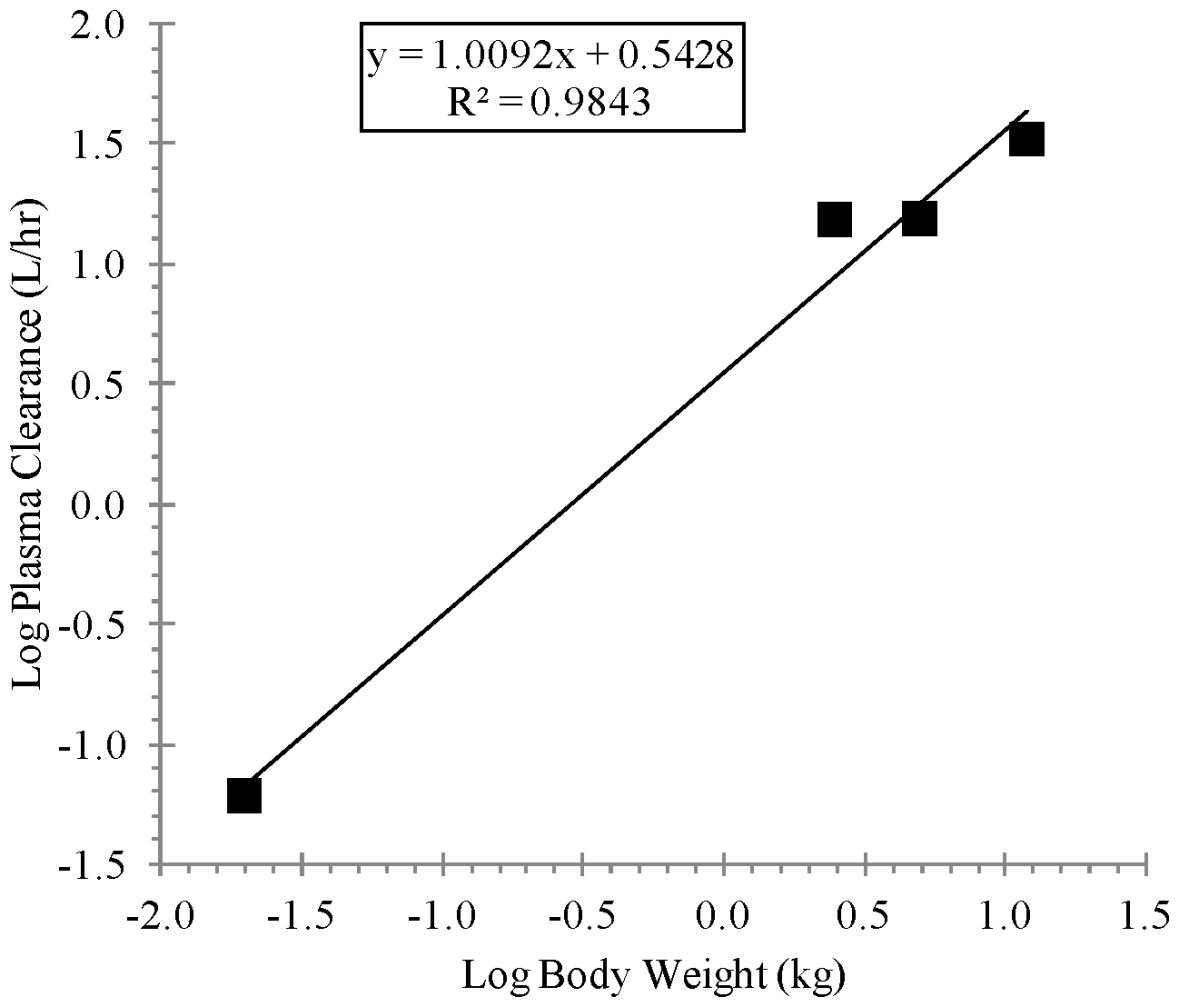
Linear regression analysis of log-transformed plasma clearance for BALB/c mice, New Zealand white Hra: (NZW) SPF albino rabbits, cynomolgus monkeys and beagle dogs versus log-transformed corresponding animal body weight, following oral administration of ST-246 by gavage at a dose of 2000, 100, 100 and 30 mg/kg, respectively.

**Figure 7:**
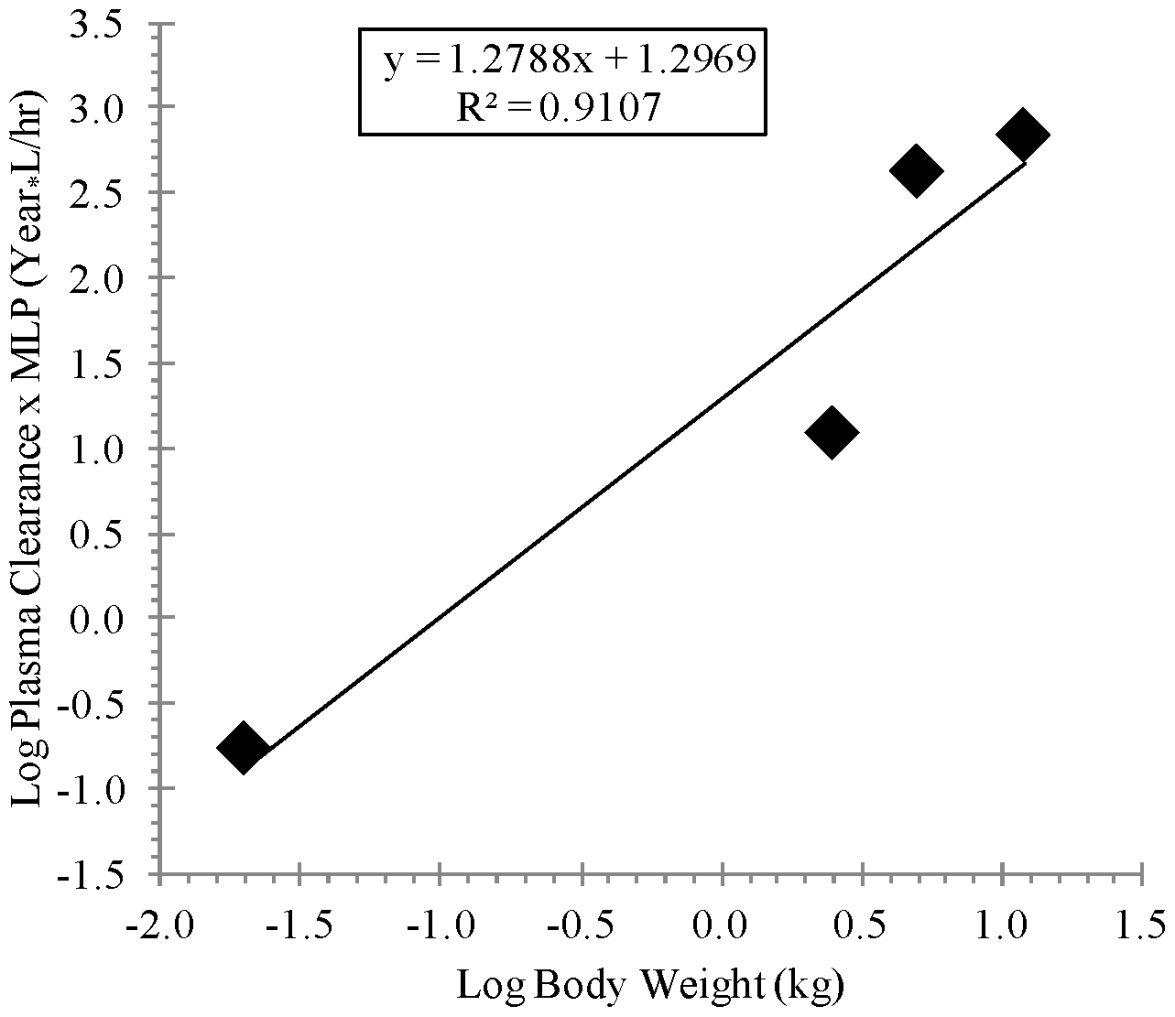
Linear regression analysis of log-transformed plasma clearance multiplied by corresponding maximum life span potential (MLP) for BALB/c mice, New Zealand white Hra: (NZW) SPF albino rabbits, cynomolgus monkeys and beagle dogs versus log-transformed corresponding animal body weight, following oral administration of ST-246 by gavage at a dose of 2000, 100, 100 and 30 mg/kg, respectively.

**Figure 8:**
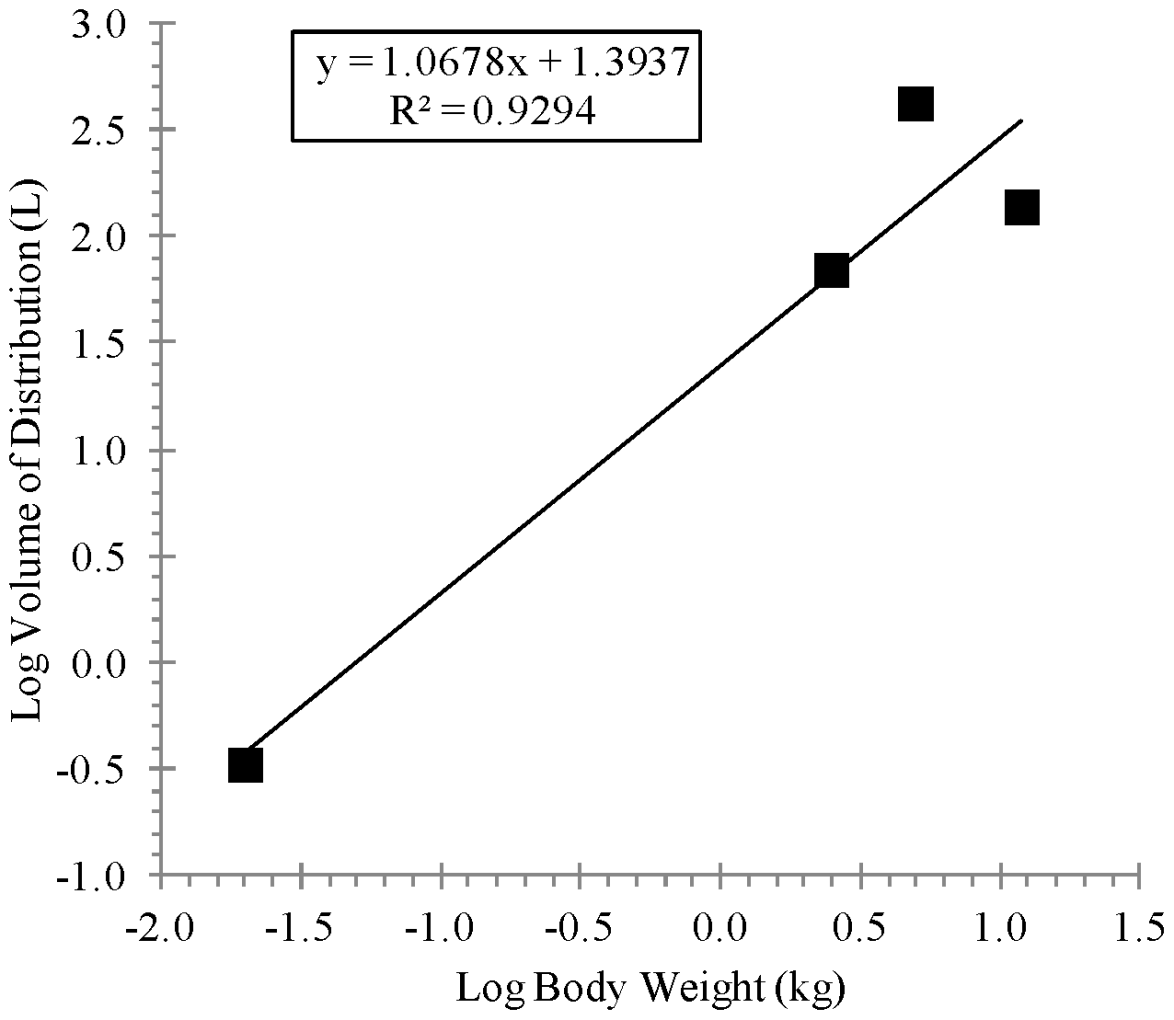
Linear regression analysis of log- transformed apparent volume of distribution for BALB/c mice, New Zealand white Hra: (NZW) SPF albino rabbits, cynomolgus monkeys and beagle dogs versus log-transformed corresponding animal body weight, following oral (gavage) administration of ST-246 by gavage at a dose of 2000, 100, 100 and 30 mg/kg, respectively.

Table 3 shows the allometric coefficient, exponent, and the coefficient of determination values derived from linear regression analysis of the of the log-transformed animal Cl/F or V_D_/F versus the corresponding log-transformed animal body weight as presented in Figures 6 and 7 for Cl/F and Figure 8 for V_D_/F. As presented in Table 3, the regression analysis showed strong correlation based on the coefficients of determination (R-squared values) for the Cl/F, (0.9843 and 0.9107 for simple and maximum life span potential (MLP)-corrected allometry, respectively) and for V_D_/F, (0.9294 for simple allometry). Also, as presented in Table 3, the allometric exponent derived from simple allometric analysis was 1.0092 for Cl/F and 1.0678 for V_D_/F and that derived from MLP-corrected allometry for Cl/F was 1.2788. Table 4 shows the observed human pharmacokinetic estimates at dose levels of 400, 600 and 800 mg and scaled Cl/F estimates of 254.02 L/h and 51.35 L/h by simple and MLP-corrected allometry, respectively, and scaled V_D_/F estimate of 2311.5 L by simple allometry. Also, as shown in Table 4, human terminal half-life values of 6.31 h (using the simple allometry Cl/F and V_D_/F estimates) and 31.20 h (using the simple allometry V_D_/F and the MLP-corrected allometry Cl/F estimates) were determined according to the relation t_1/2_ = In2_*_(V_D_/F)_scaled_/(Cl/F)_scaled_.

**Table 3:** Allometric coefficient, allometric exponent and coefficient of determination values from linear regression analysis of pharmacokinetic parameters from BALB/c mice, New Zealand white Hra: (NZW) SPF albino rabbits, cynomolgus monkeys and beagle dogs following repeat oral administration of ST-246 by gavage for 7 consecutive days in New Zealand white Hra: (NZW) SPF albino rabbits and beagle dogs and 28 consecutive days in BALB/c mice and cynomolgus monkeys.

Parameter	Allometric Coefficient	Allometric Exponent	Coefficient of Determination
Cl/F (L/h)^♀^	3.49	1.0092	0.9843
Cl/F (L/h)Φ	19.81	1.2788	0.9107
V_D_/F (L)^φ^	22.78	1.0678	0.9294

♀ & φ = Values from simple allometry.

Φ = Values from MLP-corrected allometry.

**Table 4:** Observed versus scaled human plasma pharmacokinetics, recommended and given FIH dose levels for ST-246.

Parameter		Scaled	400 (mg)^h^	600 (mg)^i^	800 (mg)^j^
Cl/F (L/h)		254.0^a^	37.5±14	48.0±25	36.5±7
Cl/F (L/h)		51.4^b^			
V_D_/F (L)		2311.5^c^	1248.2±588	1355.6±790	1081.0±459
t_1/2_ (h)		6.3^d^	25.8±11	24.3±15	20.7±8
t1/2 (h)		31.2^e^			
Extraction Ratio		0.756^f^	0.111	0.143	0.109
Extraction Ratio		0.153^g^			
Recommended ST-246 Dose (mg) Determined from Scaled Human Plasma Clearance				ST-246 Dose Levels (mg) administered in Healthy Adult Human Volunteers	
485¶				400, 600 & 800	

Extraction ratio = Cl/F/Cardiac Output.

¶ = First-in human (FIH) dose calculated by a slight modification (no safety factor applied) [26] of the pharmacologically guided approach provided in the United State Food and Drug Administration (USDFA) draft guidance [27] and expressed as follows:

Dose (mg) = Animal AUC (h_*_µg/mL) × scaled human Cl/F (L/h).

Where the applied AUC is the lowest observed systemic exposure value among the animal species utilized in this evaluation.

a = Plasma clearance scaled by simple allometry.

b = Plasma clearance scaled by MLP-corrected allometry.

c = Apparent volume of distribution scaled by simple allometry.

d = t_1/2_ calculated from the simple allometry human plasma clearance (Cl/F) and simple allometry human apparent volume of distribution (V_D_/F) using the equation: t1_/2_ = In2*(V_D_/F)_scaled_/(Cl/F)_scaled._

e = t_1/2_ calculated from the MLP-corrected allometry human plasma clearance (Cl/F) and simple allometry human apparent volume of distribution (V_D_/F) using the equation: t_1/2_ = In2*(V_D_/F)_scaled_/(Cl/F)_scaled._

f = Extraction ratio calculated from simple allometry human plasma clearance (Cl/F).

g = Extraction ratio calculated from the MLP-corrected allometry human plasma clearance (Cl/F).

h = Human pharmacokinetic estimates from a double-blind, randomized, placebo-controlled study following single daily oral administrations of ST-246 (in capsule form) at a dose of 400 mg for 14 days in non-fasted healthy adult human volunteers.

i = Human pharmacokinetic estimates from a double-blind, randomized, placebo-controlled study following single daily oral administrations of ST-246 (in capsule form) at a dose of 600 mg for 14 days in non-fasted healthy adult human volunteers.

j = Human pharmacokinetic estimates from a double-blind, randomized, placebo-controlled study following single daily oral administrations of ST-246 (in capsule form) at a dose of 800 mg for 21 days in non-fasted healthy adult human volunteers.

In addition, Table 4 shows the human extraction ratio values of 0.111, 0.143 and 0.109 calculated from the observed Cl/F at the 400, 600 and 800 mg dose levels, respectively, 0.153 determined from the MLP-corrected allometry Cl/F and 0.756 determined from the simple allometry Cl/F estimates. Also, the human extraction ratio calculated from the MLP-corrected allometry Cl/F and presented in Table 4, was more proximal to the observed human values of (at all of the different administered dose levels) compared to that determined from the simple allometry Cl/F. In addition, the first-in-humans (FIH) dose of 485 mg, calculated from the MLP-corrected allometry Cl/F estimate, showed reasonable proximity to the given low and median dose levels of 400 and 600 mg, respectively, as listed in Table 4.

## Discussion

Plasma pharmacokinetics of the anti-orthopoxvirus agent, ST-246, was evaluated following repeat-dose oral administrations by gavage, to four different animal species, namely, BALB/c mice, New Zealand White Hra:(NZW)SPF albino rabbits, cynomolgus monkeys and beagle dogs. The animal plasma clearance (Cl/F) estimate was scaled by simple and MLP-corrected allometry and the apparent volume of distribution (V_D_/F) estimate scaled by simple allometry, in order to determine human Cl/F and V_D_/F.

The lowest mean time to reach peak plasma concentration (T_max_) value of 0.83 h was observed for the dog and the highest value of 3.25 h for the monkey. Also, the dog showed a 2- to 4-fold greater dose-normalized peak plasma concentration (C_max_) compared to the other species. The highest dose-normalized area under the curve (AUC) was observed for the mouse, which was 2-fold greater than that for the rabbit and monkey both of which, by approximation, showed the lowest dose-normalized AUC. This observed species-specific difference in the dose-normalized C_max_ and AUC could be due to differences in gastrointestinal transit time and/or first-pass effect. The estimated Cl/F was observed to increase across species, from 0.05 L/h for mouse to 42.52 L/h for dog. Except for the dog, the terminal half-life estimates showed a modest increase from mouse to monkey despite the significant increase in the corresponding Cl/F estimates across these species. This observation is due to the fact that the terminal half-life is a hybrid parameter, which is also influenced by V_D_/F and as can be seen in Table 1, the V_D_/F increased significantly from mouse to monkey.

The low terminal half-life observed for the dog is consistent with the very high Cl/F and low V_D_/F estimates for this species, as presented in Table 1. In addition, the dog showed a relatively high extraction ratio as listed in Table 2, indicating that this species has approximately a 6-, 2-, and 3-fold greater ability to clear ST-246, compared to the mouse, rabbit and dog, respectively.

Interspecies allometric scaling is one approach used for scaling preclinical pharmacokinetics in order to predict human pharmacokinetics necessary for dose selection in first-in-human (FIH) studies. Mahmood and Balian have reported that three or more preclinical species is adequate for reliable scaling of Cl/F by allometry [28]. In this current study, the Cl/F and V_D_/F estimates from four species namely, mouse, rabbit, monkey and dog, were scaled to determine human Cl/F and V_D_/F, which were then used to calculate human terminal half-life and extraction ratio estimates. It has been reported that the allometric exponent, depending on the combination of species used in the scaling process, could range anywhere from 0 to 1 and sometimes even higher [29], [30]. The simple allometric exponent for Cl/F, as shown in Table 3, was slightly higher than the generally accepted baseline physiological range of 0.6 to 0.8 while that for V_D_/F was very consistent with the accepted range of 0.8 to 1.0. Based on the “Rule of Exponents” (RoE) [20], [22], the Cl/F multiplied by the maximum life span (MLP) correction approach was applied in this study to improve the human Cl/F determination, since the allometric exponent derived from the simple allometry plot was 1.0092, which approximates to 1.0.

The equation, t_1/2_ = In2*(V_D_/F_scaled_)/(Cl/F_scaled_) has been utilized to indirectly determine human terminal half-life from the allometrically scaled human Cl/F and V_D_/F estimates [22], [31], [32]. As shown in Table 3, the simple and MLP-corrected allometry produced a terminal half-life of 6.31 h and 31.22 h, respectively.

Generally, for FIH studies, the Cl/F estimate is the parameter of most interest in dose selection. To establish good safety margin in a FIH study, a relatively lower Cl/F is required to determine a safe dose. Hence, the MLP-corrected allometry-derived human Cl/F of 51.35 L/h (with a corresponding extraction ratio of 0.153 and a terminal half-life of 31.20 h) showed greater proximity to the observed human estimates at the different administered dose levels (as shown in Table 4) compared to the simple allometry-derived Cl/F of 254.0L/h (with a corresponding extraction ratio of 0.756 and a terminal half-life of 6.31 h) and therefore was utilized in the determination of a recommended starting dose in humans.

Also, since terminal half-life is directly related to V_D_/F, it goes to suggest that the large allometrically determined human V_D_/F (which is approximately 6-fold higher than the highest V_D_/F estimate, which was observed for the monkey) in this evaluation, should produce a relatively longer terminal half-life. Hence, the terminal half-life of 31.20 h calculated from the MLP-corrected allometry Cl/F appeared more reasonable and in fact, showed greater proximity to the observed human estimates than the value of 6.31 h determined from the simple allometry Cl/F, as presented in Table 4. In addition, the closeness of the MLP-corrected allometry Cl/F to the observed human estimates is supported by the closeness of the human extraction ratio (calculated from the MLP-corrected allometry Cl/F) to those determined from the observed Cl/F at the different dose levels administered in the adult human volunteers.

Interspecies scaling by allometry has enjoyed broader application in the prediction of human pharmacokinetics from animal data and is becoming an attractive and useful strategy in the determination of FIH dose [33]. In this evaluation, the calculated FIH dose of 485 mg from the MLP-corrected allometry Cl/F and the lowest animal AUC was in reasonable agreement with the low and median dose levels of 400 mg and 600 mg, respectively, administered in healthy adult human volunteers as shown in Table 4.

In summary, this evaluation shows a species-specific difference in the Cl/F for ST-246 following repeat oral administration and observed to increase across the animal species from mouse to dog. The overall shallow increase in terminal half-life across species, despite the corresponding increase in Cl/F from mouse to human can be accounted for by the significant increase in V_D_/F across these species.

Finally, the human MLP-corrected allometry CL/F, terminal half-life (calculated from the MLP-corrected allometry Cl/F and the simple allometry V_D_/F values) as well as the recommended FIH dose (determined from the MLP-corrected allometry Cl/F) are in close proximity to the corresponding observed values presented herein for healthy adult human volunteers. Hence, this current evaluation shows that not only is interspecies scaling of animal pharmacokinetics useful in the prediction of human pharmacokinetics, it is also valuable in dose selection for FIH trials.
